# A Review of Dr. Wallace and the Railway Surgeons, on Spinal Concussions

**Published:** 1894-07

**Authors:** R. M. Swearingen

**Affiliations:** Austin, Texas


### TEXAS MEDICAL JOURNAL.

ESTABLISHED JULY, 1885.

PUBLISHED MONTHLY.—SUBSCRIPTION $2.00 A YEAR.

VOL. X. AUSTIN, JULY, 1894. NO. 1.

For Texas Medical Journal.

### A REVIEW OF DR. WALLACE AND THE RAILWAY SURGEONS, ON SPINAL CONCUSSIONS.

BY R. M. SWEARINGEN, M. D., AUSTIN, TEXAS.

Read before the Austin District Medical Society, at its meeting, June 21, '94.

AN article in the September number of the *Texas Sanitarian*, by D. R. Wallace, M. D., LL. D., of Waco, entitled "Spinal Concussion and John Eric Erichsen," is worthy of greater consideration than is usually given to such papers. The high character of its author,—the immense interests involved in the subject matter discussed, and the grave charges inferentially made against one of the greatest surgeons of which "this world holds record," call at least for a fair and honest investigation, on the part of the medical profession.

The doctor commences the article referred to in these words: "It has been in my mind a kind of floating, unfixed purpose for years,—in fact since I fell in with the book,—to embrace some suitable occasion to call professional attention to its teaching. *Of their mischievous tendency a glance satisfied me upon reading the first six lectures.* An incident, of recent occurrence, and to be related in the sequel, emphasizing the purpose formed upon reading the first part of the book more than twenty years ago, aroused my languid resolution, and resulted in this hasty paper."

The incident that gave cohesive force to that floating, half fixed purpose, was a book found by Dr. Wallace in the room of a lady,—who was the plaintiff in a suit for damages against a railway company. I think it best, however, to let the doctor tell it in his own dramatic style. "The writer has been called upon,'' says he, ''within the last three years, to testify (as an expert) in three suits instituted for damages, for injuries claimed to have been received in a railway collision. All palpable frauds; all the claimants had been coached by suggestions from Erichsen's book. While at the house of one of them where I had gone to examine the condition of the patient, I saw lying upon the rude mantel a suspicious looking book bound in paper. I am a bit singular about books. It is natural for me to look at them when I see them lying around. I said the book looked suspicious. It did. There were some rather odd, red lines, I felt sure I had seen before. I, with an affected purposeless manner, picked it up. Now, this was very rude, my looking at the book! but I could not resist the temptation to see if I was good at *guess.* On the cover was, 'Concussion of the Spine, by John Erichsen.' One of the claimants referred to. asked for $20,000, for spinal concussion with resultant paralysis, She was a hale, healthy woman, about forty years old, weighing about two hundred pounds avoirdupois. She had been laboring under this *terrible affliction* for six years, and yet, *mirabile dictu,* not the least discoverable atrophy of the completely paralyzed arm, and not a symptom of nervous derangment in sight.''

Now, we are free to confess that the doctor *is* a bit singular in looking at a book, when he sees it lying around, and that he was a little rude in taking it off the rude mantle,—and the large woman had no business studying her own case, when so distinguished a neurologist as the doctor was in reach, and where so many railway surgeons could be found who would willingly relieve her of all intellectual effort in that line,—but we must insist that all these admissions do not make out a case of fraud against the woman. It is to be regretted that Dr. Wallace did not give us a more elaborate history of that woman, and it is truly unfortunate that he did not tell the methods adopted, that enabled him to reach such positive conclusions,—in the diagnosis of conditions so very difficult to the ordinary physician.

The only pointers given by him to sustain his position that she was an *impostor,* was ''*the little book found in her room,*''—and that ''*six years after the injury there was not the least discoverable atrophy in the completely paralyzed arm, and not a symptom of nervous derangement in sight.*''

Had he gone a little further into the literature of his subject, he would have made the discovery, *that the absence of atrophy* was not incontrovertible evidence against the assumption of paralysis. The sensory ganglia, the medulla oblongata, and spinal cord, receive all sensory nerves and give origin to all motors. The medulla oblongata does not, in any essential particular, differ from the spinal cord, and is considered its cranial prolongation; but whilst the ganglionic portions of the cord are made up of centers which minister to and control locomotion,—the medulla contains the centers of deglutition and of respiration,—and may, therefore, be regarded as representing the stomato-gastric and respiratory ganglia. It is well known that injuries to the spine often produce paralysis of motion without impairment of sensation,—and sometimes paralysis of sensory nerves, without affecting the nerves of motion. It seems clear, therefore—reasoning by analogy—that an injury could also affect the motor nerves, and leave untouched the sympathetic system and the medulla oblongata. When this does occur the paralysis need not, necessarily affect the nutrition of the parts paralyzed.

We are told by Prof. Carpenter, that "there is no good reason to believe that nervous agency is essential to the processes of nutrition and secretion in animals any more than to the corresponding processes in plants." "This is a question," continues that well known physiologist, "which may be more certainly determined by *observation* than by any possible *experiment*. That these processes are very readily *influenced* by changes in the condition of the nervous system, is universally admitted, and it is the intimacy of this connection that has given rise to the idea of a relation of dependency." The very volume condemned by Dr. Wallace reports a case, germane to the issue; *one not injured by a railway* and consequently suspected of all manner of crimes and conspiracies,—but by the falling of a tree that the patient himself had felled. "The force of the blow was expended on the lower cervical spine, and a complete paralysis of motion, and of sensation of all the parts below, was the immediate result. The weight of the body became greater than before the injury, and the lower limbs retained their heat and physical development. The patient had an unusual share of mental vigor, and was fond of travel, lying on his back in his carriage. Six years after the accident, he appeared before the Green County Medical Society of New York, and asked to have his legs amputated, as he wanted the room occupied by them in his carriage for books and other articles. Both limbs were amputated near the hip-joints without the slightest pain, or the tremor of a muscle. The stumps rapidly healed, and he soon resumed his wandering life,—traveling over the greater part of the United States. The case presents two remarkable facts," says Mr. Erichsen, "first, that the stumps, completely paralyzed, would have had sufficient vitality to have healed by first intention, and second, that the legs had in no way wasted in the *six years*, but had retained their normal physical development."

The physiological conditions that permitted the lower limbs of the young man to retain their normal physical development for six years, may have been present in the woman, and prevented the "least discoverable atrophy in her completely paralyzed arm." But through deference to my scholarly old friend, and for the sake of argument, we will admit that she was an impostor,—in the hands of corrupt doctors and shysters, for the sole purpose of robbing a railway corporation. We will go further, and add, that such attempts have often been made in this country, and will doubtless continue to be repeated, until the millennium, but in the name of justice, we ask, *where does John Eric Erichsen come in as particeps criminis?* He has written a book, and with marvelous accuracy described a condition more frequently met with after railway accidents than after injuries from other causes, and a popular name has been given to it, so simple and appropriate, that all men recognize the character of the injury, by its name alone. Looked at from a scientific standpoint, it matters little who reads the book, or how many millions of dollars have been taken from railway companies by its teachings. The subject that most concerns the medical man, and all other humanitarians, is far above and beyond the narrow channel of dollars and damages. *It is the simple question of truth! Did the great surgeon write of things that had no existence when he described that class of injuries? Was it the adroit work of a mischief-maker, to encourage impostors, reward the unscrupulous, or was it a revelation and a truth?*

Is there such a thing as spinal concussion; and are railway accidents from their very nature, more liable to cause them, than any other kind of accident?

*Concussion* means commotion, *to shake together*, and spinal concussion is thus defined: "The severe agitation, shaking, shock or general disturbance of the minute parts of the spinal cord." "The *primary* effects of these concussions are probably due to molecular changes in structure; the *secondary*, are of an inflammatory character dependent on retrogressive changes, such as softening."

"Four pathological conditions are embraced under the term concussion of the spine. 1st. A *jar*, disordering to a greater or less degree the cord, without any perceptible lesion. 2d. *Compression* of the cord, slowly produced by extravasation of blood. 3d. *Compression* of the cord by inflammatory exudations of serum, lymph or pus, within the spinal canal, and 4th. *Chronic alterations* of the structure of the cord itself, as the result of impairment of nutrition,—consequent on the occurrence of on eor the other of the preceding pathological states."

The charge or statement so often made, that spinal concussion, and its resultant sequelæ was unknown to medical science before the era of railways, is untrue.

Dr. Maty, in 1766, reported in the "*Medical Observations and Inquiries*," the case of Count De Lardat, a French officer, who was thrown from his carriage over a steep bank. He was bruised some, but walked to the nearest town, which was a considerable distance. "Thence he pursued his journey, and it was not until the sixth day that he was let blood on account of the injury to the shoulder and hand. He went through a campaign, but in about six months he began to find impediments to the utterance of certain words, and in the movement of his left arm. He was compelled to leave the army on account of the palsy." Sir Charles Bell, the highest authority of his time, in the *Surgical Observations*, published 1816, relates two interesting and typical cases,—one where the symptoms were immediate, and the other where they developed slowly. Prof. Gross, before Mr. Erichsen first delivered his now celebrated lectures, tells us that "concussion of the cord is produced by falls upon the head, back, feet, or nates, and that the severity of the effect is usually in proportion to the directness of the injury. That the most violent case he ever saw was in an elderly gentleman, caused by a fall upon the buttocks from a height about ten feet down upon the floor." Let us here consider the question anatomically, and see why that kind of accident, or fall, would be liable to produce the character of injury described. The cord is encased in a long, bony tube of short articulations, and has comparatively little flexibility. It is well protected from blows made at right angles to the column, and is not often hurt by such blows, unless the bony casement is fractured, or the external parts show some contusion. A severe jar, however, that strikes the column at either extremity, gives a better opportunity for an exclusively intra-vertebral injury. The force of the blow is expended *longitudinally*, and on account of the mobility of the cord within its channel, the medullary substance receives molecular momentum throughout its whole length. This molecular momentum in some cases has been sufficient to even rupture the piamater, and produce a hernia of the medullary substance. Confronted by these facts it is difficult to understand how any one can doubt the possibility or probability of molecular disturbances, when exposed to such blows. I will not reflect upon the intelligence of my hearers by adducing other arguments to sustain the theory of spinal concussion.

Assuming, then, that they do exist, the corollary is inevitable that railway collisions offer more opportunities for them than do all other casualties.

The crash and jar of an engine and train, abruptly stopped when running at the rate of forty miles an hour,—that shatters cars into splinters, that dashes passengers to and fro like shuttle-cocks, in all conceivable positions, giving blows and wrenching spinal articulations in every direction,—is the place, above all others, to find molecular injuries to brain, and spine. The difficulty of immediately discovering this class of injuries, and of discriminating between the malingerers and those actually hurt, should make us guarded and conservative,—but cannot be urged as an argument against their existence.

I am not surprised to see the chiefs of railway companies opening their batteries upon Mr. Erichsen and the so-called "railway spine." A careful perusal of the papers read before "The National Association of Railway Surgeons," since its first meeting, will suggest ulterior aims and purposes,—possibly not suspected by the casual observer. The majority of its members meet to discuss subjects pertaining to railway surgery, and to advance, by honest methods, the art and science of medicine. To all such the true physician should be in perfect sympathy. There are others, we think, who are inspired by less praiseworthy objects, and in this class will be found some of the strongest men among them. They are the "chiefs (not all, however,) of the hospital departments" for great railway systems. These are the men who formulate rules for the guidance of subordinates, furnish the schedule of charges to be made for the services rendered by them to employes, pay them their pitiful fees, mould their opinions in harmony with the new dispensation, teach them how to be experts in all manner of railway injuries, particularly the railway spine, and, above all things, to store their minds with useful knowledge, when called upon to give testimony in suits for damages.

I do not mean to convey the impression that the subordinate surgeons thus taught and drilled, will ever intentionally bear false witness, or in any manner do violence to the most stainless conscience. The object of that corporation school of surgery is to organize a corps of doctors who think alike; who believe Gross and Erichsen teach dangerous and mischievous doctrines; and who are always ready for duty when called upon to give pointers to attorneys, or give evidence in courts of justice.

Dr. W. B. Outten, of St. Louis, one of the chiefs, and an ex-president, I think, of the Association, occupies a high seat in this modern school of surgery, and might be designated as the Professor of "Theory and Practice." In the *Galveston News* of May 11th he gives some racy theories on the causes of railway concussions. It is interesting to note how ingeniously he can turn switches and side track facts that might prove detrimental to railway interests. He says: "The most typical injuries occurring upon the railway occur to the railway *employe.* His injuries are always far more violent than to the passenger. It can be shown," continues the doctor, "that in 22,929 injuries by employes, only eight cases of this trouble (railway spine) occurred among them, or one in 2400, while we can show that one in every sixty-five passengers injured have this trouble. *That when a wreck upon a railway train occurs near a large city, that you invariably have railway spine, simply for the reason that the neurologists or nerve doctors are always present in the cities, while we can show that twenty times the number of accidents occurring upon a road away from a popular center, never have them.*"

What wicked, unscrupulous fellows those town doctors must be! The thought never seems to have drifted into Dr. Outten's well-disciplined mind that the physicians of towns and cities, who are unbiased by official influences, and untrained by high authorities, whose lives are devoted to all humanity instead of a special interest, are as liable to be correct in their diagnoses as are the commissioned doctors of railway corporations. This railway chief does not tell us how the statistics were gathered that exhibit the remarkable disproportion of spinal injuries, between passengers and employes. One in every sixty-five passengers injured by railroads have spinal trouble, and only one in every twenty-four hundred of employes are so afflicted. In twenty-two thousand nine hundred and twenty-nine employes injured, *only eight cases* of that trouble are on record. When we remember that railway surgeons, as a rule, are the only ones who attend the injured employes, it is remarkable that a single case was found with that most obnoxious of all diseases. The probabilities are that those eight must have, in some inscrutable manner, escaped from railway hospitals and been treated by unpretentious, old-fashioned practitioners. With the lights before us now, it is safe to assume that had either one of the eight cases been registered on the hospital books of any of the railway systems of America, within the last year, we would find opposite their respective names either "*traumatic psychosis*" or "*hypochondriasis.*" Railway surgeons keep the records that make statistics, and they are certainly experts in the business. With my own limited opportunities for seeing that class of injuries, I now have under treatment a typical case of so-called railway spine, in the employment (as brakesman) of the Houston & Texas Central railway. He is a very intelligent colored man, who had probably never heard of spinal concussion, nor has he been trained by town-cultured neurologists for a special purpose. In a collision he was thrown violently from the flat car, upon which he stood, to the ground. He was bruised some, and for a few minutes insensible, but no serious injury could be discovered by Dr. Watts, the physician who first saw him, except a paralysis of the left leg. I was several times asked by Dr. Watts to visit the man, but failed to do so until three weeks after the the accident. At that time he presented a well marked case of spinal concussion, without the least evidence of external injuries of any kind having been inflicted upon the muscles or bones of the spinal column. The concussion had evidently been greatest about the middle of the spine and downward. There was a partial paralysis of the left leg, paralysis of the bladder, and hyperæsthesia. The foot of the affected leg was considerably swollen from impaired circulation. The spasmodic twitching, tingling sensation, expression on the face of pain and anxiety, were all present to indicate an unquestionable case of spinal concussion. Had Dr. Outten himself seen the unfortunate man, he might have been persuaded to add one more name to the list of employes whose spinal column had felt the shock of railway collisions.

A good, strong backbone is a fine thing to have, particularly in a railroad employe, and the great chiefs who control the hospital departments, seem to venerate that important part of the human anatomy, and believe it capable of enduring tremendous strains. To illustrate their superb confidence in its ability to endure, I respectfully refer to a case reported by me several years ago. A section hand was thrown from a hand-car, near Manor, Texas. Dr. Gregg, a well-informed physician of the place, was called, and found a broken spine. Under the rules of the hospital service, physicians called to accidental injuries inflicted upon employes, had to report by wire to the hospital authorities, and receive instructions to *render the needful attention*, or the account for the services would not be paid. Temporary dressings, to give immediate comfort, were made, and the message sent to headquarters. In place of orders for the doctor to continue in charge and exercise his judgment as to the time for sending his patient to the hospital, an imperative command was given to the agent of the company to "send the man on first train to the Houston Infirmary." I have no information as to who gave the order, but as it was obeyed, the presumption is legitimate that it came from some one in authority.

The attending surgeon was not notified of the intention to remove his patient, and of course had no opportunity to support the broken back by splints, or plaster dressing. As might have been expected by any one familiar with spinal injuries, long before the train that bore him reached its destination, death, in mercy, came to the sufferer's relief.

The official who gave the order of removal was doubtless ignorant of the man's physical condition, *but it was his duty to know the character of an injury before sending a telegram that had all the force, the consequences and responsibility of a death warrant.*

Not only the chiefs were heard from, at the last convocation of National Railway Surgeons, on the subject of railway spine, but a lawyer (a member of the Association) from New York, armed cap-a-pie with technical lore, leaped into the arena, and shivered a lance on the helmet of the mighty Erichsen.

Judge Clark Bell, the editor of the *Medico-Legal Journal*, read a lengthy paper on the subject. "Railway Spine," says this most learned judge, "is the Nemesis of modern surgery. Invented by one of the most clever English surgeons *as a means of procuring* enormous verdicts from railway corporations in accident cases, it has baffled both railway surgeons and counsel, and, vampire like, sucked more of the blood of corporate bodies than all other causes combined." I will quote a few sentences from this brilliant medico-legal writer, to show the exuberant fancy and spectacular rhetoric of an eloquent railway lawyer, when he sees that awful vampire sucking the life blood from inoffensive corporate bodies. Still on the railway spine, he continues: "Unknown before the era of steam, it is a plant sprouted on English soil, which has exhibited phenomenal growth in its infancy,—has developed in the last half of the present century into a tree that, like the banyan, has sent out branches that have taken root in the soil of all countries where civilization has carried her standard. Unknown, absolutely, among the savages" (that is remarkable, as the savages are so well informed on all other subjects pertaining to nervous disorders), "not found away from railways, it has been an incubus and parasite upon that magnificent growth, the modern railway, which, like the ivy on the oak, has nearly strangled the trunk by its contaminating influence."

Now, who would have believed that a little volume, based upon scientific truths and describing pathological conditions known to exist, could have equalled in its blighting influence the combined forces of war, pestilence, and famine. *Invented to promote fraud and mischief, it is a remorseless vampire, a banyan tree, an incubus, an insatiable parasite!*

The great Englishman little dreamed of the fate in store for him, in this far-away western world, when he launched those fourteen lectures on the sea of surgical literature. Yes, Judge Bell was right in one statement, if in no other. Mr. Erichsen did invoke a kind of Nemesis, that throws its arms around poor broken, paralyzed, maimed humanity, and with even handed justice holds the scales for the weak and helpless against the rich and strong. In the eyes of the sage of Waco, and the railway surgeons of America, he may have committed a lasting wrong, and they may herald their theories to the uttermost bounds of the earth,—but they will never dim the lustre of so noble a life. If all other books written by him were lost, his name and fame would go down the ages on that single volume, "Concussion of the Spine."

---

How dear to our heart is
  Cash on subscription,
When the generous subscriber
  Presents it to view;
But the man who don't pay—
  We refrain from description,
For, perhaps, gentle reader,
  That man might be you.

—*Chatham (N. Y.) Courier.*

